# EPR Study of KO_2_ as a Source of Superoxide and ^•^BMPO-OH/OOH Radical That Cleaves Plasmid DNA and Detects Radical Interaction with H_2_S and Se-Derivatives

**DOI:** 10.3390/antiox10081286

**Published:** 2021-08-13

**Authors:** Anton Misak, Vlasta Brezova, Miroslav Chovanec, Karol Luspai, Muhammad Jawad Nasim, Marian Grman, Lenka Tomasova, Claus Jacob, Karol Ondrias

**Affiliations:** 1 Biomedical Research Center, Department of Molecular Physiology, Institute of Clinical and Translational Research, Slovak Academy of Sciences, Dúbravská Cesta 9, 84505 Bratislava, Slovakia; anton.misak@savba.sk (A.M.); marian.grman@savba.sk (M.G.); lenka.tomasova@savba.sk (L.T.); 2 Institute of Physical Chemistry and Chemical Physics, Faculty of Chemical and Food Technology, Slovak University of Technology in Bratislava, Radlinského 9, 81237 Bratislava, Slovakia; vlasta.brezova@stuba.sk (V.B.); karol.luspai@stuba.sk (K.L.); 3 Biomedical Research Center, Department of Genetics, Cancer Research Institute, Slovak Academy of Sciences, Dúbravská Cesta 9, 84505 Bratislava, Slovakia; miroslav.chovanec@savba.sk; 4 Division of Bioorganic Chemistry, School of Pharmacy, University of Saarland, D-66123 Saarbruecken, Germany; jawad.nasim@uni-saarland.de (M.J.N.); c.jacob@mx.uni-saarland.de (C.J.)

**Keywords:** KO_2_, antioxidants, EPR spectra simulation, ^•^BMPO-OOH spin adduct, superoxide, radical, hydrogen sulfide, selenium-derivatives, cleavage DNA

## Abstract

Superoxide radical anion (O_2_^•−^) and its derivatives regulate numerous physiological and pathological processes, which are extensively studied. The aim of our work was to utilize KO_2_ as a source of O_2_^•−^ and the electron paramagnetic resonance (EPR) spin trapping 5-*tert*-butoxycarbonyl-5-methyl-1-pyrroline *N*-oxide (BMPO) technique for the preparation of ^•^BMPO-OOH and/or ^•^BMPO-OH radicals in water solution without DMSO. The method distinguishes the interactions of various compounds with ^•^BMPO-OOH and/or ^•^BMPO-OH radicals over time. Here, we show that the addition of a buffered BMPO-HCl mixture to powdered KO_2_ formed relatively stable ^•^BMPO-OOH and ^•^BMPO-OH radicals and H_2_O_2_, where the ^•^BMPO-OOH/OH ratio depended on the pH. At a final pH of ~6.5–8.0, the concentration of ^•^BMPO-OOH radicals was ≥20 times higher than that of ^•^BMPO-OH, whereas at pH 9.0–10.0, the ^•^BMPO-OH radicals prevailed. The ^•^BMPO-OOH/OH radicals effectively cleaved the plasmid DNA. H_2_S decreased the concentration of ^•^BMPO-OOH/OH radicals, whereas the selenium derivatives 1-methyl-4-(3-(phenylselanyl) propyl) piperazine and 1-methyl-4-(4-(phenylselanyl) butyl) piperazine increased the proportion of ^•^BMPO-OH over the ^•^BMPO-OOH radicals. In conclusion, the presented approach of using KO_2_ as a source of O_2_^•−^/H_2_O_2_ and EPR spin trap BMPO for the preparation of ^•^BMPO-OOH/OH radicals in a physiological solution could be useful to study the biological effects of radicals and their interactions with compounds.

## 1. Introduction

Superoxide anion radical (O_2_^•−^) is essential for the life of aerobic organisms, regulating numerous physiological and pathophysiological processes. When an overproduction of O_2_^•−^ occurs and/or the antioxidant defense is deficient, oxidative stress may develop, leading to oxidative damage in many biological systems [1,2]. Therefore, the production, biological interactions, and control of the O_2_^•−^ concentration have all been intensively studied both in vivo and in vitro.

For experiments involving radical O_2_^•−^ reactions, O_2_^•−^ can be generated using various methods [3], e.g., enzymatically by xanthine/xanthine oxidase [4,5], which is a system too complex for some types of O_2_^•−^ radical models. It can also be generated by ionizing radiation or by dissolving superoxide salts in aprotic solvents, e.g., KO_2_ in anhydrous DMSO; however, DMSO may not be desirable in some biological experiments [3,6]. On the other hand, KO_2_ dissolved in an aqueous solution is a simple source of O_2_^•^^−^, but its lifetime is in the range of milliseconds [7]. For these reasons, it would be practical and appropriate to have an easily accessible O_2_^•^^−^ source that could be used in physiological solutions. Therefore, the aim of our work was to examine KO_2_ as a suitable source of O_2_^•^^−^, which can be used to react with cyclic nitrone 5-tert-butoxycarbonyl-5-methyl-1-pyrroline-N-oxide (BMPO), generating superoxide (^•^BMPO-OOH) or hydroxyl (^•^BMPO-OH) radical adducts for further studies in aqueous systems.

The electron paramagnetic resonance (EPR) spin trapping technique, using BMPO, is useful to study short-lived O_2_^•−^ and hydroxyl (HO^•^) radicals [4,5,8,9,10,11,12,13,14,15]. By trapping O_2_^•−^, BMPO produces relatively stable ^•^BMPO-OOH spin adducts, which can be further studied as a potential model for organic hydroperoxides. A saturated KO_2_ solution in DMSO has been used as a source of O_2_^•−^ to form ^•^BMPO-OOH. The presence of DMSO is convenient in studies with water-insoluble compounds, but it could be a limitation for biological studies.

Exogenously added and endogenously produced hydrogen sulfide (H_2_S) influences many physiological and pathologic processes, including oxidative stress, by reacting with reactive oxygen species [16,17]. Organoselenium compounds have been known to serve as multifunctional agents. Such compounds have been shown to provide an excellent anti-cancer activity by serving as pro-oxidants that ultimately alter the redox homeostasis of cancer cells, leading to apoptosis. Many biological activities have been associated with organoselenium compounds, ranging from simple antioxidants and antimicrobials, to rather complicated immuno-modulatory, anti-inflammatory, and anti-nociceptive effects [18]. As organoselenium compounds have also been known for their interaction with radicals, the compounds RSe-1 and RSe-2 were employed in this study.

Therefore, in the present work, a new procedure for the preparation of relative stable ^•^BMPO-OOH/OH adducts from KO_2_ as a source of O_2_^•−^ without the use of DMSO as a solvent is described. The ^•^BMPO-OOH radical was mostly observed at pH ~6.5–8.0. ^•^BMPO-OOH/OH cleaved plasmid DNA and ^•^BMPO-OOH/^•^BMPO-OH total radical concentrations and their proportion were influenced by H_2_S- and selenium-containing organic compounds.

## 2. Materials and Methods

### 2.1. Chemicals, EPR Sample Preparation, and Measurement

Na_2_S was purchased from DoJindo (SB01, Munich, Germany), and the other chemicals were purchased from Sigma-Aldrich (Steinheim, Germany). Na_2_S dissociates in the solution and reacts with H^+^ to yield H_2_S, HS^−^, and traces of S^2−^. We used H_2_S to refer the total mixture of H_2_S, HS^−^, and S^2−^. A stock solution of 100 mmol L^−1^ Na_2_S was prepared in argon-bubbled ultrapure deionized H_2_O, and was stored at −80 °C for a few days and used immediately after thawing.

**Scheme 1:**
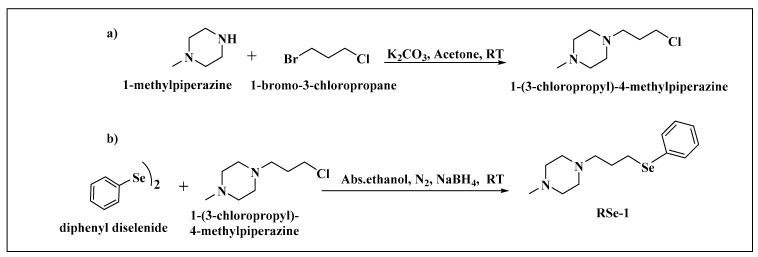
Synthesis of compound RSe-1. (**a**)The first step of synthesis involves the alkylation of 1-methylpiperazine with 1-bromo-3-chlropropane to produce 1-(3-chloropropyl)-4-methylpiperazine in the presence of K_2_CO_3_ and acetone at room temperature. (**b**) 1-(3-chloropropyl)-4-methylpiperazine is subsequently reacted with diphenyl diselenide in an inert environment of N_2_, in the presence of NaBH_4_ and absolute ethanol at room temperature to obtain RSe-1 as final product.

**Scheme 2:**
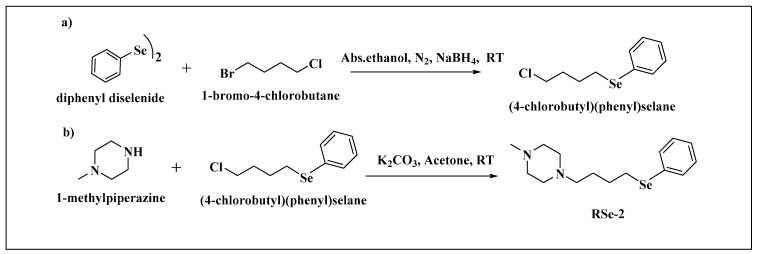
Synthesis of compound RSe-2. (**a**)The first step of synthesis involves the Se-alkylation of 1-bromo-4-chlorobutane with diphenyl diselenide in an inert environment of N_2_, in the presence of NaBH_4_ and absolute ethanol at room temperature to obtain (4-chlorobutyl)(phenyl)selane. (**b**) In the second step (4-chlorobutyl)(phenyl)selane is reacted with 1-methylpiperazine in the presence of K_2_CO_3_ and acetone at room temperature to obtain RSe-2 as final product.

Two selenium derivatives, 1-methyl-4-(3-(phenylselanyl)propyl)piperazine (RSe-1) and 1-methyl-4-(4-(phenylselanyl)butyl)piperazine (RSe-2), were obtained according to the synthesis routes shown in Scheme 1; Scheme 2. For the compound RSe-1, methyl piperazine was alkylated, employing 1-bromo-3-chloropropane in the presence of anhydrous K_2_CO_3_ and acetone as a solvent. The reaction was performed at room temperature (RT) and the progress of the reaction was controlled by thin layer chromatography. The crude product was used for Se-alkylation in absolute ethanol under a nitrogen atmosphere at room temperature (Scheme 1). For the compound RSe-2, Se-alkylation of 1-bromo-4-chlorobutane was performed under a nitrogen atmosphere and in absolute ethanol at RT. The crude product was then reacted with methyl piperazine in the presence of anhydrous K_2_CO_3_ and acetone at 50 °C (Scheme 2) [19]. The structure and purity of the final compounds were confirmed via spectroscopic and chromatographic methods. The final products were converted into crystalline hydrochloric salts to improve their solubility in water.

The spin-trapping agent, 5-*tert*-butoxycarbonyl-5-methyl-1-pyrroline *N*-oxide (BMPO, DoJindo B568, Munich, Germany), was dissolved in ultrapure deionized H_2_O (100 mmol L^−1^), stored at −80 °C, and used after thawing. For the EPR samples, a buffer consisting of 50 mmol L^−1^ sodium phosphate and 100 µmol L^−1^ diethylenetriaminepentaacetic acid (DTPA; pH 7.4; 37 °C) was used. Powdered KO_2_ (Sigma-Aldrich 278904, Steinheim, Germany) in amounts of 0.5–1 mg was weighed into tubes just before being used. As the dissolution of KO_2_ in a phosphate buffer shifts from pH to alkaline values, an adequate volume of 1 mol L^−1^ HCl was added to the buffer containing BMPO (20 mmol L^−1^) to get the required final pH after the addition to KO_2_. Twelve seconds after adding the BMPO-containing phosphate solution to the powdered KO_2_, compounds Na_2_S in H_2_O and RSe-1 or RSe-2, prepared in the buffer, were added. The pH in the samples was measured by pH indicator paper (Ahlstrom-Munksjö, Germany) with an accuracy of ±0.2. The detailed procedure for the sample preparation for EPR measurement is described in the Supplementary Materials. The sample was transferred to a standard cavity aqueous EPR flat cell (WG 808-Q, Wilmad-LabGlass, Vineland, NJ, USA), and the EPR spectra were measured as described previously [6]. The first EPR spectrum was recorded 100 ± 15 s after the addition of the BMPO solution to powdered KO_2_. The sets of individual EPR spectra of the BMPO spin adducts were recorded as 15 or 30 sequential scans of 42 s each, with a total acquisition time of 11 or 22 min, respectively. The EPR spectra of the BMPO spin adducts were measured on a Bruker EMX spectrometer (Rheinstetten, Germany) essentially as in our previous study [6]. All of the EPR spectra were recorded at 37 °C. The spectra were simulated using the EasySpin program working on the MatLab platform [20]. The concentration of ^•^BMPO-OOH and ^•^BMPO-OH radicals was evaluated from a double integral of their EPR spectra and compared with the spectra of 20 µmol L^−1^ 4-hydroxy-2,2,6,6-tetramethylpiperidine-1-oxyl radical (TEMPOL, Fluka 42777, Munich, Germany), which was used as a reference sample. To compare the potency of the investigated compounds for decreasing the overall trapped radical concentration, a double integral of the total EPR spectra intensity of the BMPO adducts was evaluated. The concentration of H_2_O_2_ was measured by a Fluorimetric Hydrogen Peroxide Assay Kit (MAK165, Merck, Bratislava, Slovakia) according to the manufacturer’s instructions. The concentrations of KO_2_ and the BMPO/KO_2_ mixture were diluted 1000 and 2000 times, respectively, before the measurement of H_2_O_2_.

### 2.2. Plasmid DNA (pDNA) Cleavage Assay

A pDNA cleavage assay with the use of the pBR322 plasmid (N3033 L, New England BioLabs, Inc., Ipswich, MA, USA) was performed as reported previously, with some modifications [6]. All of the final samples contained the studied compounds and 0.2 μg of pDNA in 20 µL of the sodium phosphate buffer (25 mmol L^−1^ sodium phosphate, 50 μmol L^−1^ DTPA; pH 6.5, 7.4, 8.0, or 8.5). The procedure for the sample preparation of the pDNA cleavage assay is described in detail in the Supplementary Materials. Likewise, in the EPR sample preparation, the phosphate buffer was adjusted by HCl to keep the final pH at the required value after being added to powdered KO_2_ (20 mmol L^−1^) (see Procedure 5 in Supplementary Materials). In the procedure focused on the KO_2_−BMPO interaction, the powdered KO_2_ (20 mmol L^−1^) was dissolved by 10 mmol L^−1^ BMPO in a 45 mmol L^−1^ phosphate buffer adjusted by HCl, then vortexed for 10 s, and 10 µL of the mixture was added to a 10 µL solution of pDNA in a 5 mmol L^−1^ phosphate buffer (see Procedure 6 in Supplementary Materials). The pH conditions were also standardized in the cleavage assay through the addition of 0.5 mol L^−1^ NaOH (for pH 6.5) or 0.1/0.125 mol L^−1^ HCl (for pH 8.0/8.5) to the pDNA solution to reach pH 7.4 for all of the samples (see Procedure 7 in Supplementary Materials).

For the preparation of the BMPO/KO_2_ mixture, in which, according to EPR experiments, ^•^BMPO-OOH/OH radicals were not formed, powdered KO_2_ (20 mmol L^−1^) was dissolved in a 50 mmol L^−1^ phosphate buffer adjusted by HCl, vortexed for 10 s, and then BMPO (10 mmol L^−1^) was added. As in the previous experiments, 10 µL of the mixture was added to 10 µL of the pDNA solution (see Procedure 8 in Supplementary Materials). All of the final samples contained 5 mmol L^−1^ BMPO and 10 mmol L^−1^ KO_2_ in the sodium phosphate buffer (25 mmol L^−1^ sodium phosphate, 50 μmol L^−1^ DTPA; pH 6.5, 7.4, 8.0 or 8.5). FeCl_2_ (150 µmol L^−1^), as a massive pDNA cleavage inductor, was used as a positive control in the assay. The resulting mixtures were incubated for 30 min at 37 °C. After incubation, the reaction mixtures were subjected to 0.6% agarose gel electrophoresis. The integrated densities of all pBR322 forms in each lane were quantified using Image Studio analysis software (LI-COR Biotechnology, Bad Homburg, Germany) to estimate the pDNA cleavage potency through the relative intensities (I_R_) of the nicked form of pDNA (see Figure S2 in Supplementary Materials).

## 3. Results

### 3.1. pH-Dependent Composition of ^•^BMPO-Adducts of (BMPO+HCl)-KO_2_ Interaction

**Figure 1:**
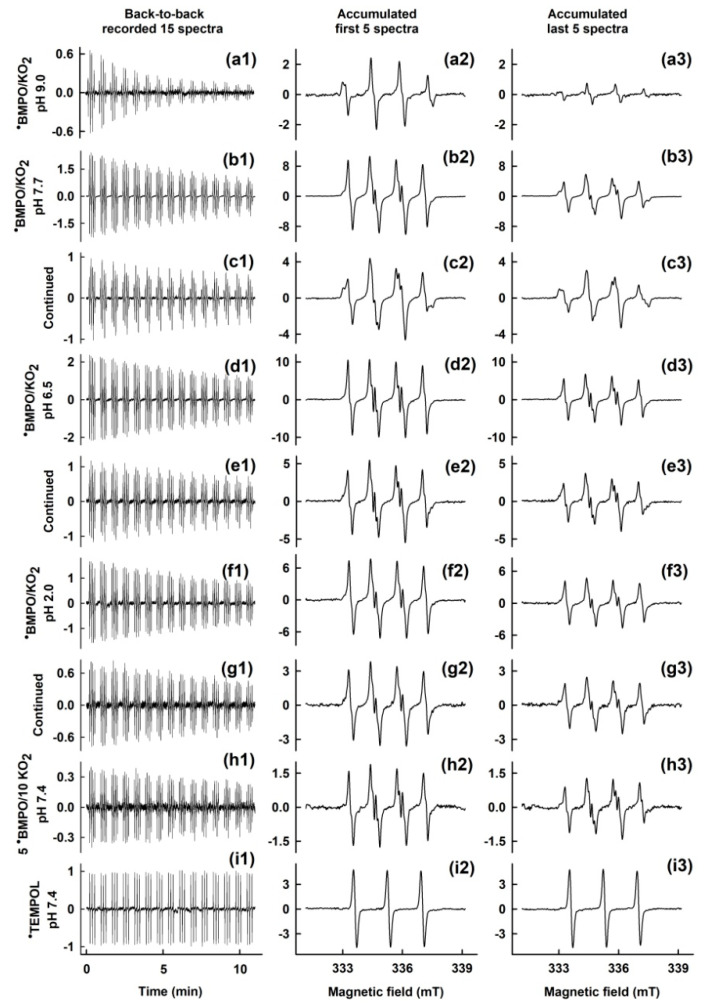
Representative pH-dependent EPR spectra of ^•^BMPO adducts after the addition of 20 mmol L^−1^ BMPO + HCl to powdered KO_2_ (final 40 mmol L^−1^ KO_2_). (**a1**–**i1**) Collection of 15 EPR spectra of BMPO adducts arranged back-to-back, each 42 s, with a starting acquisition of 100 ± 15 s after sample preparation. (**a2**–**i2**) The first to fifth accumulated spectra and (**a3**–**i3**) last five accumulated spectra. HCl was added to BMPO (20 mmol L^−1^) in a buffer for the final required pH and the solution was added to powdered KO_2_. (**a1**–**a3**) pH 9.0; (**b1**–**b3**) pH 7.7 and (**c1**–**c3**) continuation; (**d1**–**d3**) pH 6.5 and (**e1**–**e3**) continuation; (**f1**–**f3**) pH 2.0 and (**g1**–**g3**) continuation. (**h1**–**h3**) ^•^BMPO adducts after the addition of 5 mmol L^−1^ BMPO + HCl to powdered KO_2_ (final 10 mM KO_2_) at pH 7.4. (**i1**–**i3**) The EPR spectra of stable radical TEMPOL (20 µmol L^−1^). Intensities of (**a1**–**h1**) time-dependent EPR spectra and (**a2**–**h3**) the detailed spectra are approximately comparable. For details of the sample preparation, see Procedure 2 in Supplementary Materials.

**Figure 2:**
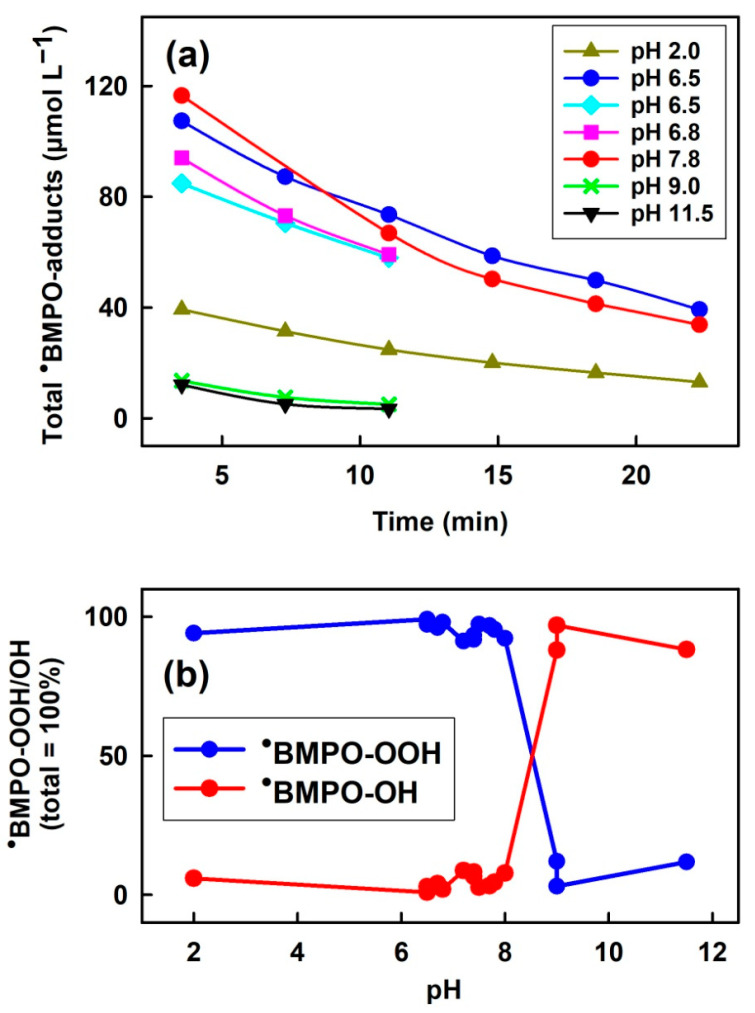
(**a**) Time and pH dependencies of the total ^•^BMPO adduct radical concentration of the BMPO + HCl/KO_2_ (20/40 in mmol L^−1^) mixture. Each point represents the average of five accumulated subsequent EPR spectra. Time starts after the addition of BMPO + HCl to powdered KO_2_. (**b**) pH-dependent normalized integral EPR intensity of individual ^•^BMPO-OOH and ^•^BMPO-OH components elucidated from the simulation of the first experimental EPR spectrum recorded 100 ± 15 s after the addition of BMPO + HCl to powdered KO_2_. Spectral simulation is described in Section 3.2. Spectral components: ^•^BMPO-OOH (blue) and ^•^BMPO-OH (red). The sum of individual BMPO adducts of the first spectra was normalized to 100%. Each pair (blue and red) of two complementary points represents the individual sample preparation.

In the control experiment, a phosphate buffer adjusted with HCl was added to powdered KO_2_ (final 40 mmol L^−1^ KO_2_) to get a final pH value of 7.4. After 10–13 s, when BMPO (final 20 mmol L^−1^) was added to the KO_2_–buffer solution, the EPR spectra of ^•^BMPO adducts were not observed (for details, see Procedure 1 in Supplementary Materials). However, when BMPO (20 mmol L^−1^) in a phosphate buffer (pH 7.4) was added to powdered KO_2_ (final 40 mmol L^−1^), EPR spectra, depending on final pH, were observed (Figure 1). After the BMPO-containing phosphate solution (pH 7.4) was added to the powdered KO_2_, the pH increased to ~10–11. Therefore, to study the pH dependence of the radical formation from KO_2_, HCl was added to BMPO in the buffer, which ensured that after the BMPO solution was added to the powdered KO_2_, the final required pH was obtained (for details, see Procedure 2 in Supplementary Materials). The intensity and shapes of the ^•^BMPO adducts spectra depended on the pH. The spectral intensity of ^•^BMPO adducts obtained at pH 9.0 decreased in time (Figure 1a1–a3). In comparison with pH 9.0, the EPR spectral intensity of the ^•^BMPO adducts at pH 7.7 was higher with a different relative abundance of individual species and slow decay of the adducts. The ^•^BMPO adducts were observed for more than 22 min at pH 7.7 (Figure 1b1–c3). A similar effect on the spectra of the ^•^BMPO adducts was also observed at pH 6.5 (Figure 1d1–e3) and pH 2 (Figure 1f1–g3). By comparison, the intensity of the EPR spectra of the ^•^BMPO adducts formed in the mixture of 5 mmol L^−1^ BMPO and 10 mmol L^−1^ KO_2_ at pH 7.4 was quantitatively lower (Figure 1h1–h3), but similar to that of the higher BMPO–KO_2_ concentration (Figure 1b1–e3). The stable nitroxide radical TEMPOL (20 µmol L^−1^) was used to determine the concentration of the radicals in the samples (Figure 1i1–i3). Using the double integrals of the TEMPOL calibration, the concentration of the total ^•^BMPO adduct radicals was high at pH 6.5–7.8 and low at pH 9.0–11.5, and decreased in time, with a halftime (pH 6.5–7.8) of approximately ~12 min (Figure 2a).

As described in detail in Section 3.2, the first recorded spectrum was simulated to obtain the individual components of ^•^BMPO-OOH (sum of conformers 1 and 2) and ^•^BMPO-OH (sum of conformers 1 and 2) at 100 ± 15 s after the sample preparation (Figure 2b). The ratio of the component population significantly depended on the pH; the portions of ^•^BMPO-OOH and ^•^BMPO-OH were ~95% and ~5%, respectively, of the whole concentration within a pH of ~2–8, and ~10% and ~90% within a pH of ~9–11.

As H_2_O_2_ is produced during the reaction of KO_2_ with H_2_O, its concentration was measured in samples containing KO_2_ at pH 7.4. H_2_O_2_ concentrations of 8.9 ± 1.4 (*n* = 4) and 10.5 ± 0.7 (*n* = 4) mmol L^−1^ were formed after the addition of buffer + HCl or BMPO + HCl to the powdered KO_2_ (final 20/40 BMPO/KO_2_ in mmol L^−1^), respectively.

### 3.2. Simulation of EPR Spectra of pH-Dependent BMPO Adducts

**Figure 3:**
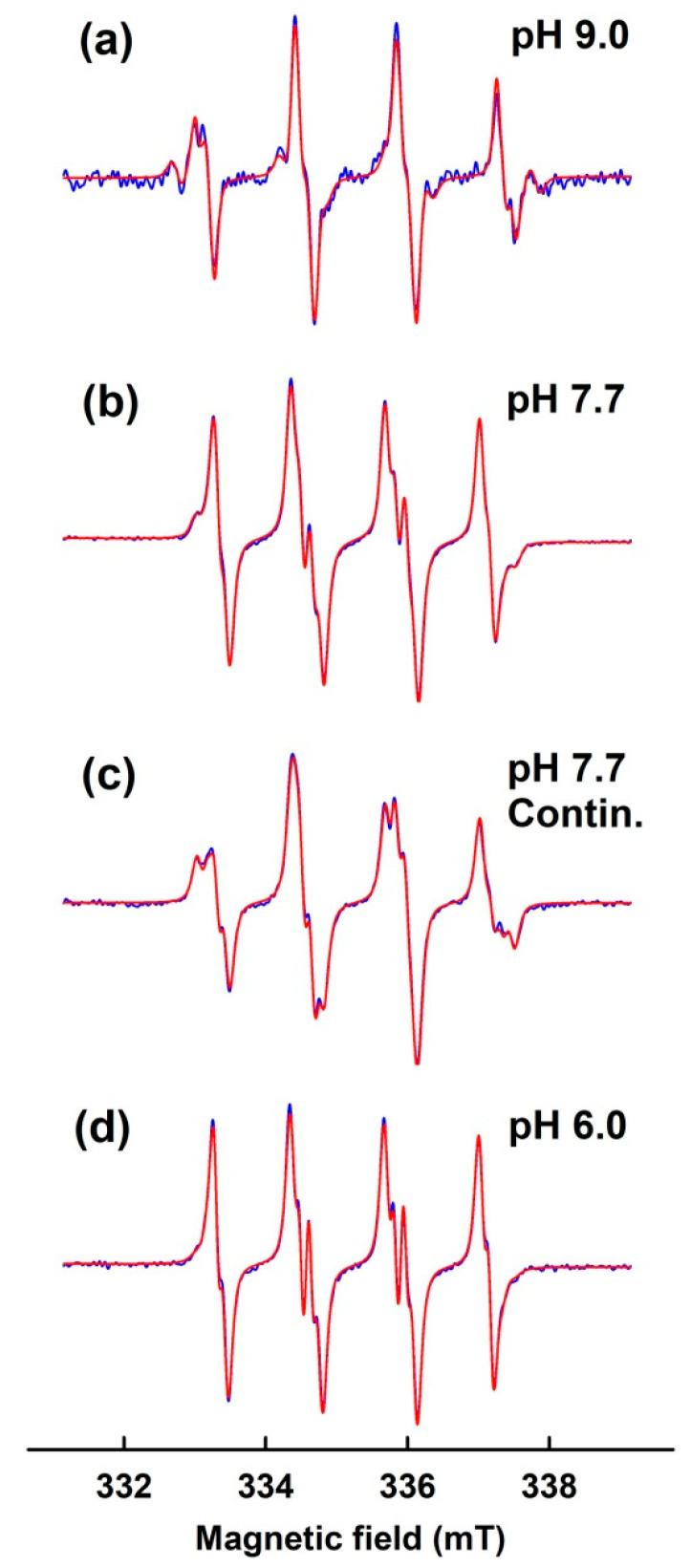
Representative normalized experimental EPR spectra of BMPO adducts along with their simulations using the hfcc summarized in Table 1. Only the experimental spectra of the sixth to tenth accumulated spectra are shown (blue, measured 5.2–8.7 min after sample preparation); the simulated spectra are red. (**a**) pH 9.0, (**b**) pH 7.7, (**c**) continued measurement during 15.7–19.2 min, and (**d**) pH 6.0.

The shapes of the EPR spectra of the BMPO adducts depended on the pH (Figure 1) and changed over time, indicating a dynamic superposition of signals corresponding to the generation of individual BMPO adducts. Therefore, we analyzed the EPR spectra by simulation, as in [6]. Two conformers each of ^•^BMPO-OOH and ^•^BMPO-OH adducts were inserted into the spin Hamiltonian calculations of the experimental spectra measured in the pH range of 2–8. However, in the EPR spectra measured in the solutions at pH ≥ 9, two additional low-intensity six-line signals were detected, which were assigned to the BMPO adduct with a carbon-centered radical (^•^BMPO-CR) [8] and ^•^BMPO-CO_2_^−^, most likely originating from the DTPA decomposition in the alkaline solutions. The simulated spectra shown in Figure 3 were calculated using the hyperfine coupling constants (hfcc) elucidated from the experimental spectra (Table 1). The comparison of the experimental and simulated spectra shows a good fit when using the hfcc. The detailed simulation analysis of the hfcc of the individual BMPO adducts evaluated from the EPR spectra measured at various pH values revealed only a negligible pH effect on the hfcc.

### 3.3. Comparison of pH-Dependent BMPO Adduct Spectra of BMPO−KO_2_ Interaction

**Figure 4:**
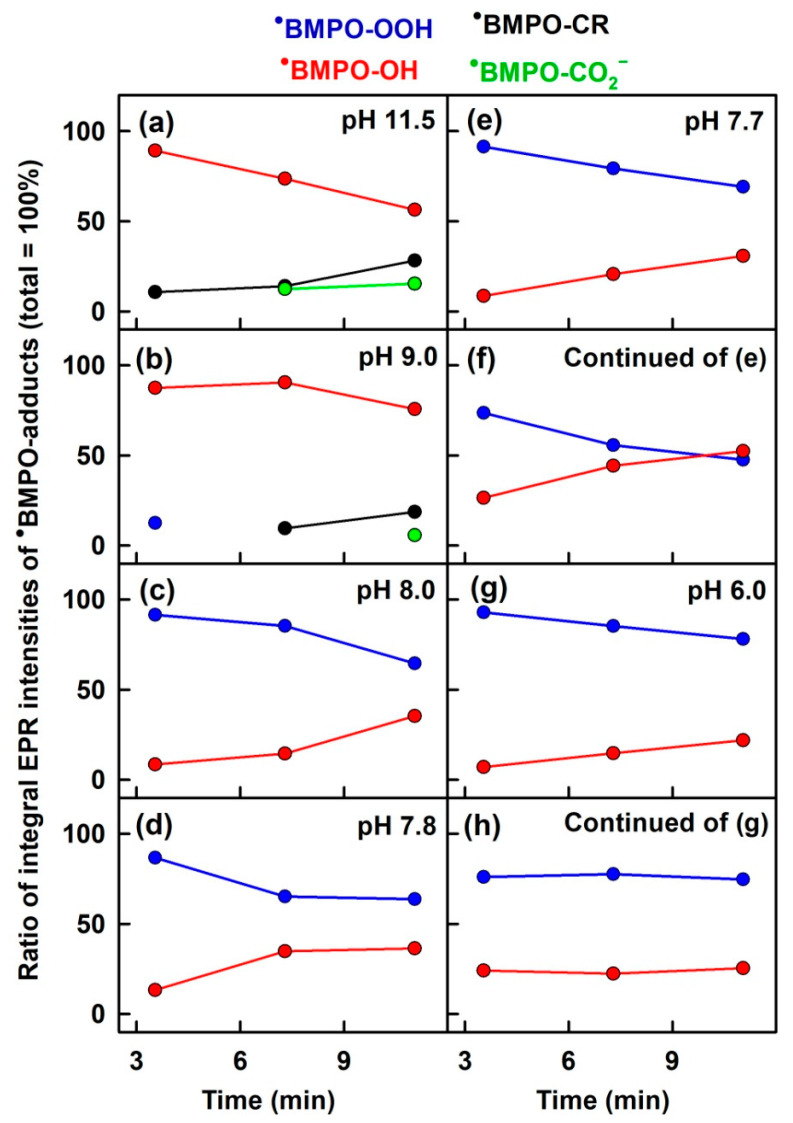
Comparison of time and pH-dependent normalized integral EPR intensity of individual BMPO adducts elucidated from the simulation of experimental EPR spectra. Spectral components: ^•^BMPO-OOH (blue), ^•^BMPO-OH (red), ^•^BMPO-CR (black), ^•^BMPO-CO_2_^-^ (green) at (**a**) pH 11.5 (pH was adjusted by NaOH), (**b**) pH 9.0, (**c**) pH 8.0, (**d**) pH 7.8, and (**e**) pH 7.7; (**f**) continued measurement of (**e**) for the next 11 min; (**g**) pH 6.0 and (**h**) continued measurement of (**g**) for the next 11 min. Each point represents the average of five accumulated subsequent EPR spectra. Time starts after the addition of BMPO + HCl to powdered KO_2_. The sum of individual BMPO adducts of accumulated spectra was normalized to 100%. For details of the sample preparation, see Procedure 2 in Supplementary Materials.

For simplicity, the relative concentrations of two conformers, ^•^BMPO-OH(1) and ^•^BMPO-OH(2), were summed and described as ^•^BMPO-OH. Analogously, ^•^BMPO-OOH(1) and ^•^BMPO-OOH(2) were summed and described as ^•^BMPO-OOH. As described in Section 3.1, the pH-dependent formation of individual ^•^BMPO-OOH and ^•^BMPO-OH components elucidated from the simulation of the first experimental EPR spectrum is shown in Figure 2b. The time dependence of the EPR intensity of the simulated components of 1–5, 6–10, and 11–15 accumulated experimental BMPO adduct spectra at different pH values is shown in Figure 4. The sum of individual ^•^BMPO adducts of the accumulated spectra at a given time was normalized to 100%. At pH 11.5, the ^•^BMPO-OH component decreased in time, and ^•^BMPO-CR and ^•^BMPO-CO_2_^−^ components were present (Figure 4a). Similarly, mostly ^•^BMPO-OH, as well as a minor abundance of ^•^BMPO-CR, ^•^BMPO-CO_2_^−^, and ^•^BMPO-OOH components, were observed at pH 9.0 (Figure 4b). However, at a lower pH (≤8.0), the ^•^BMPO-OOH component prevailed over ^•^BMPO-OH (Figure 4c,d,e,g). The relative concentration of ^•^BMPO-OOH decreased and ^•^BMPO-OH increased gradually over time at pH 7.7–8.0 (Figure 4c–e). The relative concentration of ^•^BMPO-OOH in comparison with ^•^BMPO-OH was high at pH 6.0 and relatively stable for over 11 min (Figure 4g,h).

**Figure 5:**
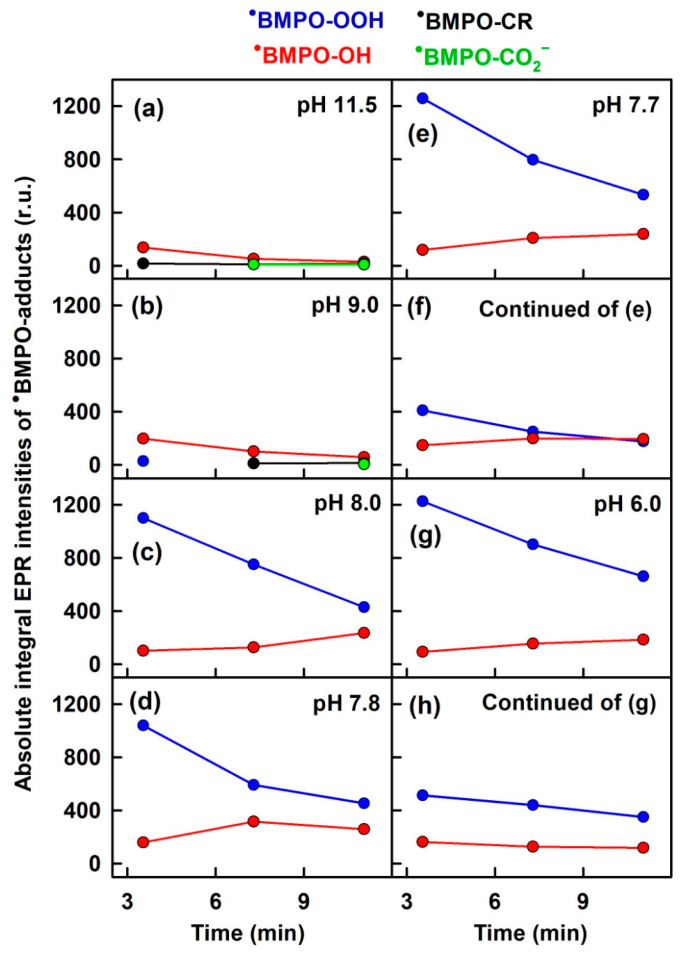
Comparison of pH-dependent absolute integral EPR intensity (total quantity of radicals in r.u.) of individual BMPO adducts elucidated from the simulation of the experimental EPR spectra. Spectral components: ^•^BMPO-OOH (blue), ^•^BMPO-OH (red), ^•^BMPO-CR (black) and ^•^BMPO-CO_2_^−^ (green) formed after dissolution of KO_2_ (final 40 mmol L^−1^) in 50 mmol L^−1^ phosphate buffer containing BMPO (20 mmol L^−1^) at (**a**) pH 11.5, (**b**) pH 9.0, (**c**) pH 8.0, (**d**) pH 7.8, (**e**) pH 7.7 and (**f**) continued measurement of (**e**), (**g**) pH 6.0 and (**h**) continued measurement of (**g**).

To compare the pH dependence of the total radicals of the ^•^BMPO adduct components, double integral intensities of the simulated spectra were applied (Figure 5). The concentration of trapped radicals of the ^•^BMPO adducts was very low at pH ~9.0–11.5 (Figure 5a,b). However, at pH ~6.0–8.0, the number of trapped radicals increased ~3–10 times, particularly because of the ^•^BMPO-OOH component (Figure 5c–h). The amount of ^•^BMPO-OOH radical components was ~10 times higher in comparison with ^•^BMPO-OH at pH ~6.0–8.0, 3.5 min after the sample preparation (Figure 5c–h). The presence of the ^•^BMPO-OOH component was still ~3 times more abundant in comparison with ^•^BMPO-OH after 22 min at pH ~6.0 (Figure 5h).

### 3.4. Effect of Na_2_S and Selenium Derivatives on ^•^BMPO-OOH/OH Radicals

**Figure 6:**
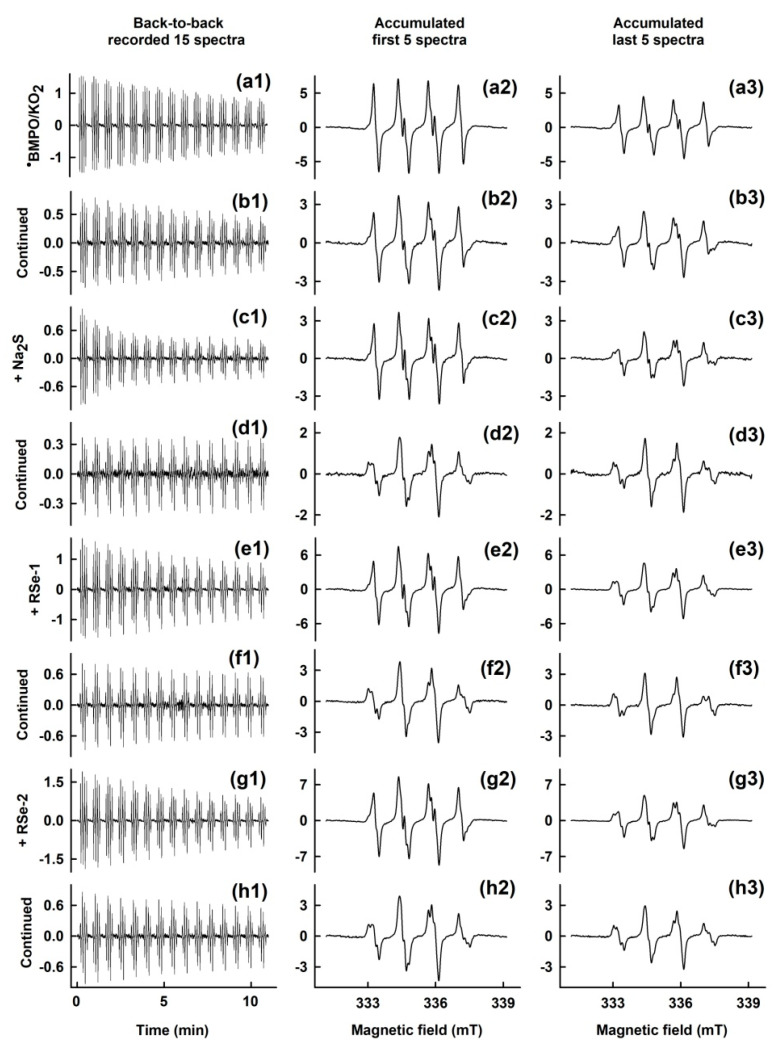
Effects of the compounds on the EPR spectra of ^•^BMPO adducts prepared through the addition of BMPO + HCl to powdered KO_2_. Final concentrations: BMPO + HCl/KO_2_ (20/40 in mmol L^−1^) in a 50 mmol L^−1^ phosphate buffer at pH 7.4. The arrangement of the spectra is the same as in Figure 1. (**a1**–**b3**) Control EPR spectra and (**c1**–**d3**) EPR spectra in the presence of 100 µmol L^−1^ Na_2_S, (**e1**–**f3**) 100 µmol L^−1^ RSe-1, and (**g1**–**h3**) 100 µmol L^−1^ RSe-2. For details of the sample preparation, see Procedure 3 in Supplementary Materials.

**Figure 7:**
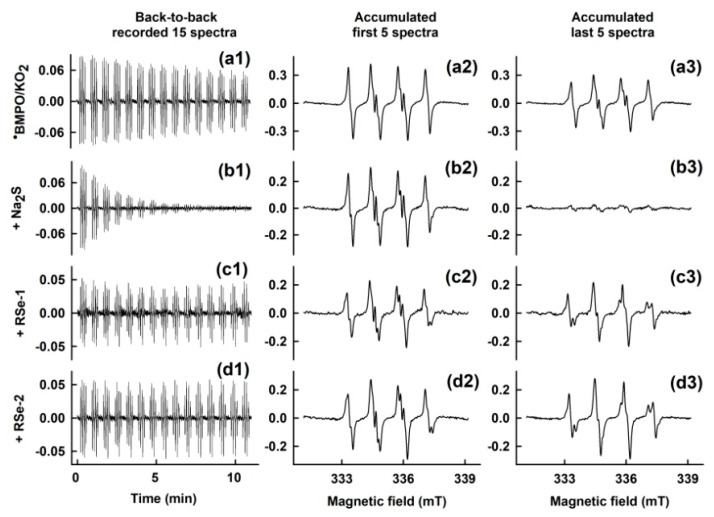
Effects of the compounds on the EPR spectra of ^•^BMPO adducts prepared through the addition of BMPO + HCl to powdered KO_2_. Final concentrations: BMPO + HCl/KO_2_ (20/40 in mmol L^−1^) in 50 mmol L^−1^ phosphate buffer at pH 6.7. The arrangement of the spectra is the same as in Figure 1. (**a1**–**a3**) Control EPR spectra and (**b1**–**b3**) EPR spectra in the presence of 100 µmol L^−1^ Na_2_S, (**c1**–**c3**) 100 µmol L^−1^ RSe-1, and (**d1**–**d3**) 100 µmol L^−1^ RSe-2. For details of sample preparation, see Procedure 3 in Supplementary Materials.

**Figure 8:**
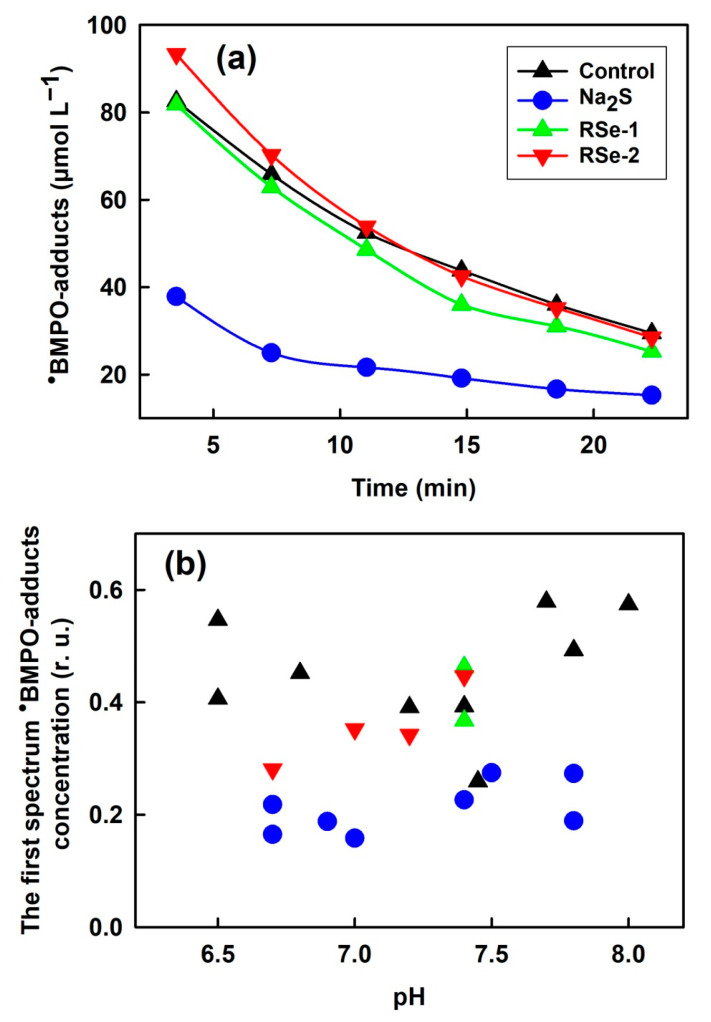
(**a**) Time dependencies of the total ^•^BMPO adduct radical concentration of the BMPO + HCl/KO_2_ (20/40 in mmol L^−1^) mixture (pH 7.4) with 100 µmol L^−1^ Na_2_S (blue), 100 µmol L^−1^ RSe-1 (green), and 100 µmol L^−1^ RSe-2 (red), as well as without any of these (black). Each point represents the average of five accumulated subsequent EPR spectra. Time starts after the addition of BMPO + HCl to powdered KO_2_. (**b**) The pH dependence of the integral EPR intensity of the total ^•^BMPO adducts (r.u.) of the first spectrum measured at 100 ± 15 s after the addition of BMPO + HCl to powdered KO_2_. Symbols have same meaning as in (**a**). Each point represents individual sample preparation.

The spectral intensity and shape of the ^•^BMPO adducts depended on the time and compounds added to the samples at pH 7.4 and 6.7 (Figure 6 and Figure 7). The spectral intensity of ^•^BMPO adducts for all of the samples decayed over time and was visible for more than 22 min (Figure 6 and Figure 7). However, at pH 6.7, the decay in RSe-1 and RSe-2 was complex, and was not further investigated in detail (Figure 7). Na_2_S (100 µmol L^−1^) decreased the total concentration of ^•^BMPO adducts in comparison with the controls (Figure 6c1–d3, Figure 7b1–b3 and Figure 8a. The effect of RSe-1 and RSe-2 on the total concentration of ^•^BMPO-adducts was not significantly different from the control spectra at pH 7.4 (Figure 8a). A comparison of the effect of the compounds on the total ^•^BMPO adduct concentration of the first recorded spectrum at pH 6.5–8.0 is shown in Figure 8b. The concentrations of ^•^BMPO adducts were high for the controls at pH 6.5–8.0. Na_2_S (100 µmol L^−1^) decreased the spin adduct concentration, whereas the effects of RSe-1 and RSe-2 were not significantly different compared with the controls at pH 7.4.

**Figure 9:**
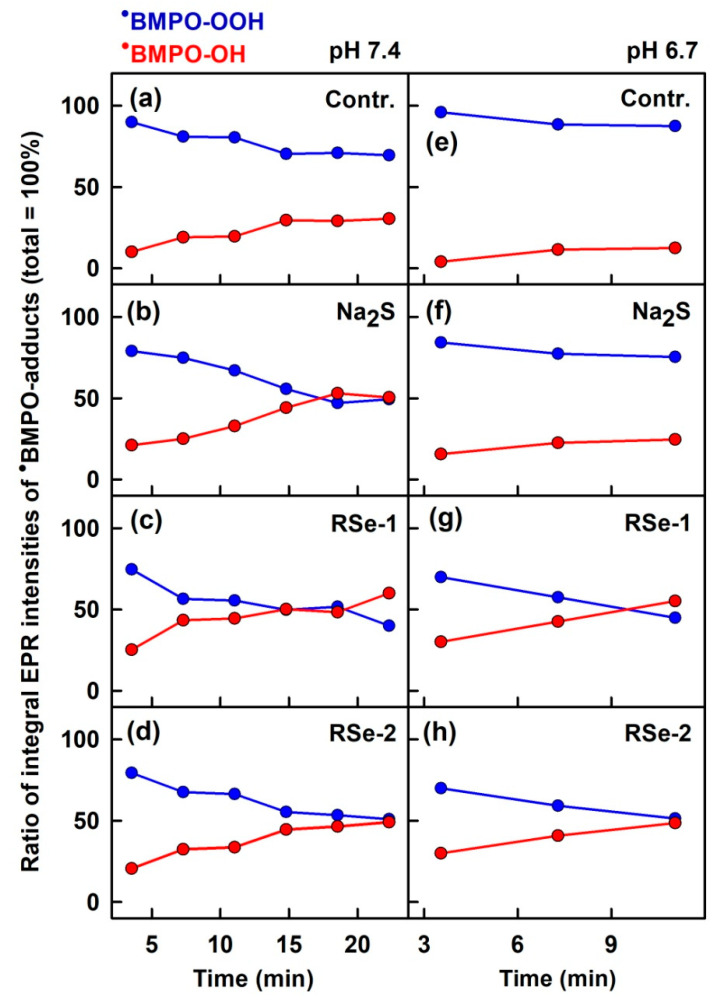
Time dependence of the integral EPR intensity normalized to 100% of the individual ^•^BMPO-OOH and ^•^BMPO-OH adducts elucidated from the simulation of the experimental EPR spectra shown in Figure 6; Figure 7, at pH 7.4 (**a**–**d**) and pH 6.7 (**e**–**h**). Time started when BMPO + HCl was added to KO_2_. Spectral components: ^•^BMPO-OOH (blue) and ^•^BMPO-OH (red). (**a**,**e**) Control; (**b**,**f**) 100 µmol L^−1^ Na_2_S; (**c**,**g**) 100 µmol L^−1^ RSe-1; (**d**,**h**) 100 µmol L^−1^ RSe-2. Each point represents the average of five accumulated subsequent EPR spectra.

The experimental spectra were simulated as previously described (Section 3.2), and revealed ^•^BMPO-OOH and ^•^BMPO-OH components (Figure 9). In the controls (pH 7.4 and 6.7), ^•^BMPO-OOH slightly decreased in time, but prevailed over ^•^BMPO-OH, which slightly increased for 22 min (Figure 9a,e). The simulation data for RSe-1 and RSe-2 (Figure 9c,d,g,h) were calculated from the experimental spectra shown in Figure 6 and Figure 7. Interestingly, RSe-1 and RSe-2, which did not change the total radical concentrations compared with control spectra at pH 7.4 (Figure 8a), significantly decreased ^•^BMPO-OOH and increased the ^•^BMPO-OH components over time, reaching equilibrium in 15–22 min at pH 7.4 (Figure 9c,d) and in 9–10 min at pH 6.7 (Figure 9g,h). Na_2_S decreased ^•^BMPO-OOH and increased the ^•^BMPO-OH components in time, similar to RSe-1 and RSe-2 at 7.4 pH, but had a minor effect at pH 6.7 (Figure 9b,f).

**Figure 10:**
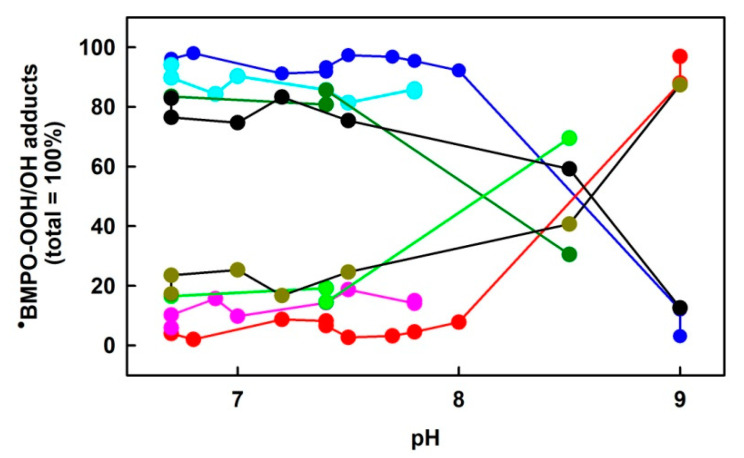
pH-dependent normalized integral EPR intensity of individual ^•^BMPO-OOH and ^•^BMPO-OH components elucidated from the simulation of the first experimental EPR spectrum recorded 100 ± 15 s after the addition of BMPO + HCl to powdered KO_2_. Spectral components: control ^•^BMPO-OOH (blue) and ^•^BMPO-OH (red) are from Figure 2b; 100 µmol L^−1^ Na_2_S (cyan and pink); 100 µmol L^−1^ RSe-1 (dark green and green), and 100 µmol L^−1^ RSe-2 (black and dark yellow). Each pair of complementary colors, ^•^BMPO-OOH and ^•^BMPO-OH, represents an individual sample preparation. The sum of the individual spectra of BMPO adducts was normalized to 100%.

The first recorded spectrum at 100 ± 15 s after the sample preparation was obtained through spectrum simulation, as described in Section 3.2 (Figure 10). The control ^•^BMPO-OOH component within pH 6.7–8.0 was ~95–98% and ^•^BMPO-OH was ~2–5% of the total ^•^BMPO adducts. At pH 9.0, the ratio was the opposite. The ^•^BMPO-OOH component within pH 6.7–8.0 decreased to ~75–90% and ^•^BMPO-OH increased to ~10–25% in the samples with Na_2_S, RSe-1, and RSe2 compared with the controls (Figure 10). The results confirm that the studied compounds started to influence the ^•^BMPO-OOH/OH radicals early after the sample preparation.

### 3.5. Composition of ^•^BMPO Adducts of BMPO−KO_2_ Mixture after Addition of HCl

**Figure 11:**
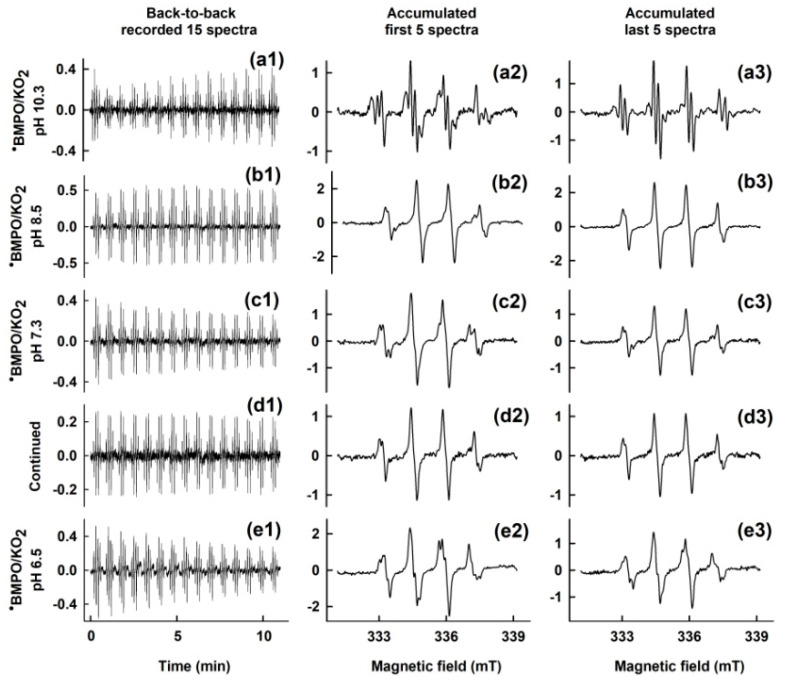
pH-dependent EPR spectra of the ^•^BMPO adduct after the addition of BMPO to powdered KO_2_, followed by the adjustment of the final pH by HCl. The arrangement of the spectra is the same as in Figure 1. BMPO in the phosphate buffer was added to powdered KO_2_ and vortexed, and 30 s later, HCl was added to adjust the final solution pH. (**a1**–**a3**) pH 10.3; (**b1**–**b3**) pH 8.5; (**c1**–**c3**) pH 7.3 and (**d1**–**d3**) continued of (**c1–c3**); (**e1**–**e3**) pH 6.5. Intensities of (**a1**–**e1**) time-dependent EPR spectra and (**a2**–**e2**,**a3**–**e3**) detailed spectra are approximately comparable. Molar ratio of BMPO/KO_2_ was 20/40 (in mmol L^−1^), except for (**a1**–**a3**), where the ratio was 56/56 (in mmol L^−1^). For details of the sample preparation, see Procedure 4 in Supplementary Materials.

**Figure 12:**
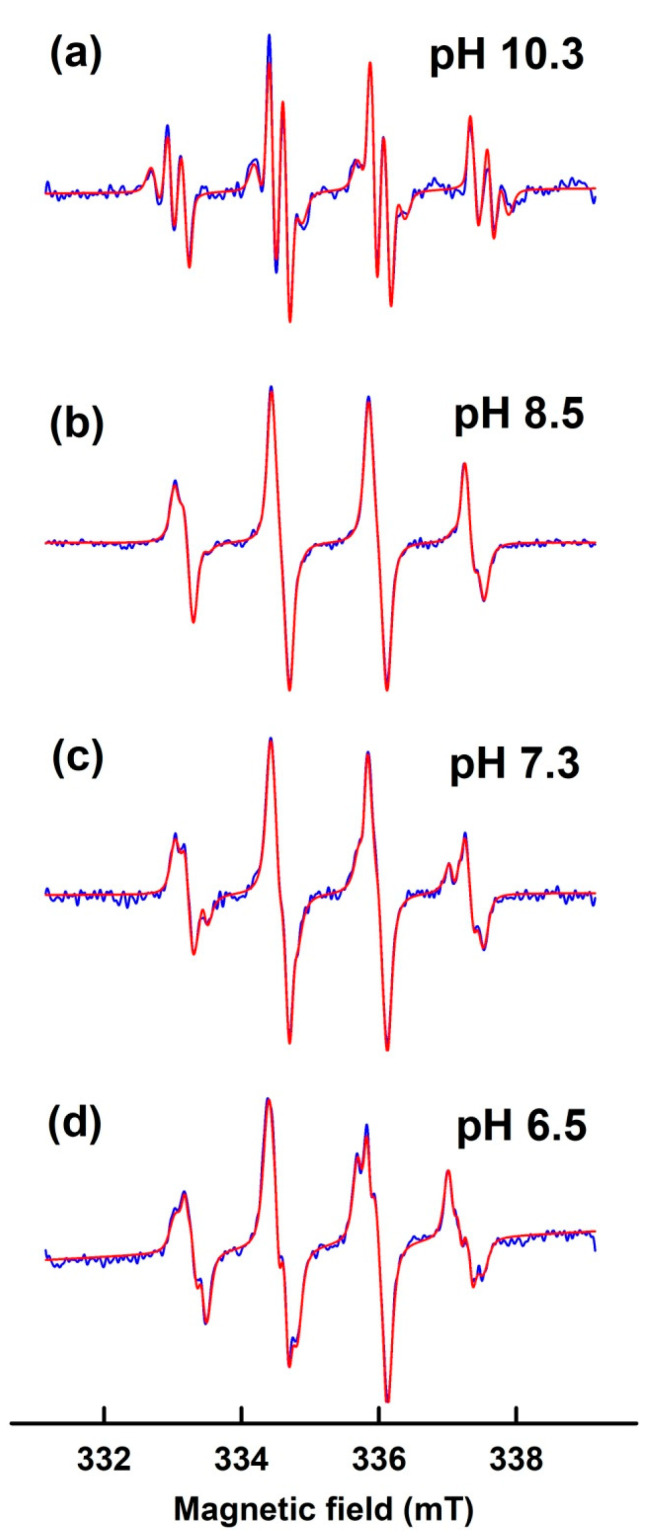
Representative normalized experimental EPR spectra of BMPO adducts along with their simulations. Arrangement of experimental and simulated spectra is the same as in Figure 3. EPR spectra at (**a**) pH 10.3, (**b**) pH 8.5, (**c**) pH 7.3, and (**d**) pH 6.5. Sample preparation: BMPO in the buffer was added to powdered KO_2_ and vortexed, and 30 s later HCl was added to adjust the final pH of the solution.

**Figure 13:**
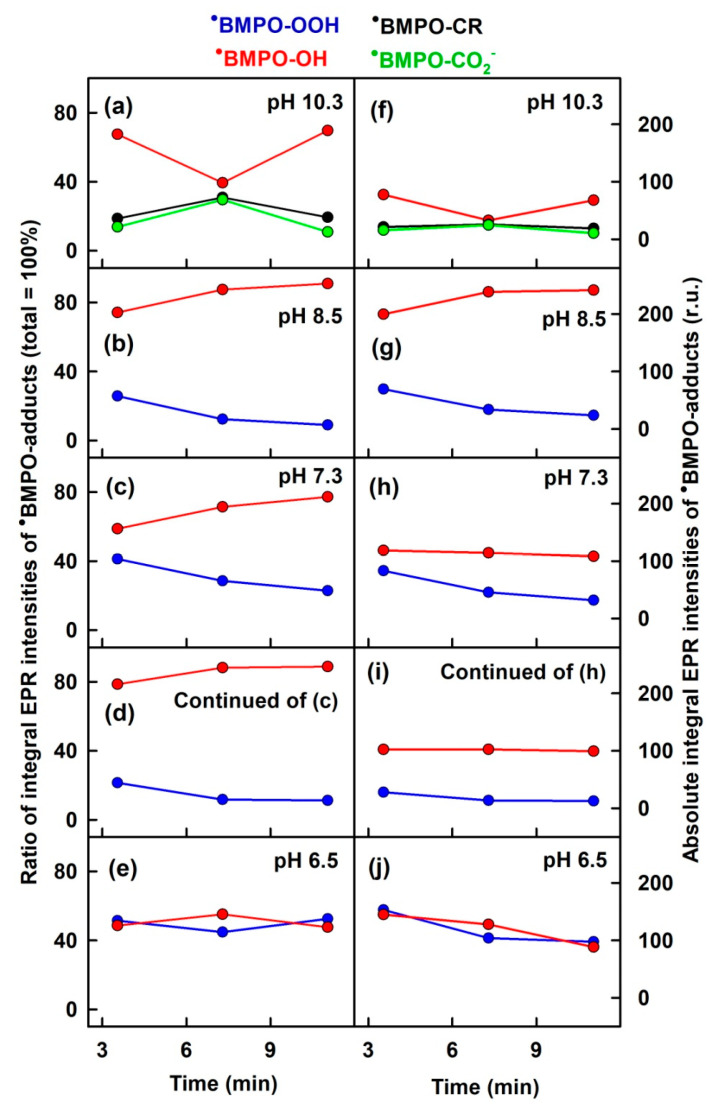
(Left column) Comparison of time- and pH-dependent normalized integral EPR intensity of individual BMPO adducts elucidated from the simulation of the experimental EPR spectra. Spectral components: ^•^BMPO-OOH (blue), ^•^BMPO-OH (red), ^•^BMPO-CR (black), and ^•^BMPO-CO_2_^−^ (green) at (**a**) pH 10.3, (**b**) pH 8.5, and (**c**) pH 7.3; (**d**) continued measurement of (**c**) for another 11 min; (**e**) pH 6.5. Each point represents the average of five accumulated subsequent EPR spectra. Time starts after the addition of BMPO to powdered KO_2_. The sum of the individual BMPO adducts of the accumulated spectra was normalized to 100%. (Right column) (**f**–**j**) Comparison of time- and pH-dependent absolute integral EPR intensity (total quantity of radicals in r.u.) of individual BMPO adducts of normalized integral EPR intensity shown in the left column.

A different procedure for ^•^BMPO adduct preparation was also used. In this procedure, the BMPO solution was added to powdered KO_2_, and 30 s later, the pH was adjusted by HCl to the required value. For details of the sample preparation, see Procedure 4 in the Supplementary Materials. The pH dependence of the BMPO adduct spectra showed significant differences (Figure 11 and Figure 12) compared with the spectra observed in Figure 1, Figure 3 and Figure 4 when HCl was added to the BMPO solution before KO_2_. The spectra depended on the pH and increased/decreased over time for more than 22 min. In order to find the spectral components of the ^•^BMPO adducts of the samples prepared by the latter procedure, the EPR spectra were simulated as in the previous cases (Section 3.2). A comparison of the experimental and simulated spectra at different pH values (Figure 12) showed that the best simulation fit was obtained using the hfcc (Table 1). The time dependence of the spectral intensity of the simulated components of BMPO adducts at different pH values is shown in Figure 13. The left and right columns show the relative and absolute proportions of the spectral components, respectively. At pH 10.3, ^•^BMPO-OH, ^•^BMPO-CR, and ^•^BMPO-CO_2_^−^ adducts (but not ^•^BMPO-OOH) were observed (Figure 13a,f). The absolute value of the ^•^BMPO adduct radicals at pH 10.3 was low compared with that at pH 6.5–8.5. At pH 7.3–8.5, the component ^•^BMPO-OH increased in time and prevailed over ^•^BMPO-OOH, which decreased in time (Figure 13b,c,g,h). At pH 6.5, both ^•^BMPO-OOH/OH components were present in approximately equally amounts (Figure 13e,j).

### 3.6. pH-Dependent Cleavage of pDNA by BMPO–KO_2_ Interaction

**Figure 14:**
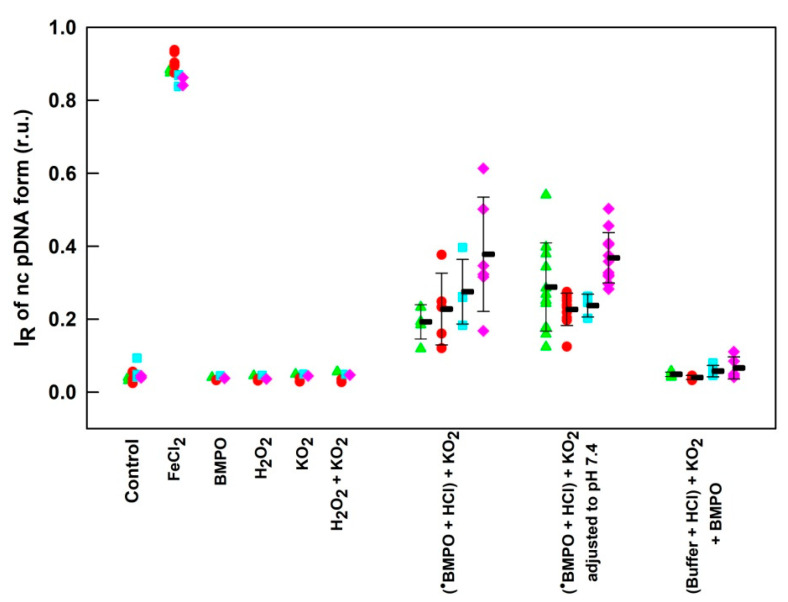
Potency of compounds to induce pDNA cleavage at pH 6.5 (green), 7.4 (red), 8.0 (cyan), and 8.5 pH (pink) in a 25 mmol L^−1^ sodium phosphate buffer and 50 µmol L^−1^ DTPA at 37 °C. Control without and with 150 µmol L^−1^ FeCl_2_; controls with 5 mmol L^−1^ BMPO, 5 mmol L^−1^ H_2_O_2_, 10 mmol L^−1^ KO_2_, and 5 mmol L^−1^ H_2_O_2_ + 10 mmol L^−1^ KO_2_. Sample (BMPO + HCL) + KO_2_: BMPO (final 5 mmol L^−1^) in a phosphate buffer adjusted by HCl (to get required pH of BMPO/KO_2_ mixture) was added to powdered KO_2_ (final 10 mmol L^−1^), then the mixture was added to the pDNA solution (see Procedure 6 in Supplementary Materials). Sample (BMPO + HCL) + KO_2_ adjusted to pH 7.4: solutions were prepared as for previous samples with various pH levels, but HCl or NaOH were added to the pDNA solution to standardize the pH to 7.4 (see Procedure 7 in Supplementary Materials). Sample (Buffer + HCl) + KO_2_ + BMPO: phosphate buffer adjusted by HCl was added to powder KO_2_ (final 10 mmol L^−1^), followed by the addition of BMPO (final 5 mmol L^−1^) 10 s later, and the mixture was applied to the pDNA solution (see Procedure 8 in Supplementary Materials). The final concentration of pDNA was 0.2 µg in 20 µL. I_R_ of nc DNA form represents the relative intensity of the nicked circular pDNA. Data represent values from individual samples. Horizontal black marks indicate means ± SD.

To know whether ^•^BMPO adducts have biological effects, the cleavage of pDNA by ^•^BMPO-OOH/OH was evaluated (Figure 14 and Figure S2). pDNA cleavage was negligible in the control buffers at pH 6.5, 7.4, 8.0, and 8.5, but significantly increased in the presence of FeCl_2_, which is known to cleave DNA [21,22]. Besides the single-stranded cleavage of the supercoiled pDNA generating the nicked circular form, FeCl_2_ was notably able to mediate double-stranded cleavage, creating linear form (Figure S2 in Supplementary Materials). The control samples containing BMPO, H_2_O_2_, KO_2_, or the mixture of H_2_O_2_ and KO_2_ did not induce pDNA cleavage. However, when BMPO + HCl was added to powdered KO_2_, producing ^•^BMPO-OOH/OH, and then applied to pDNA, significant pDNA cleavage was observed at all of the pH values studied. The values were scattered, but the pDNA cleavage potency had a tendency to increase with the increasing pH values. To confirm the involvement of pH in the observed effect, the solution of pDNA in the phosphate buffer was adjusted by NaOH or HCl to standardize the pH to 7.4 after the addition of (BMPO + HCl) + KO_2_ solutions with pH 6.5, 8.0, and 8.5. After the pH was adjusted to 7.4, the effects of the mixtures were still significant, but the pH trend was lost. Based on the EPR measurement of the radical concentration, pDNA was significantly cleaved by ^•^BMPO-OOH/OH at a concentration of ~18 µmol L^−1^, where the concentration ratio of ^•^BMPO-OOH to ^•^BMPO-OH at the beginning of the experiments was ≥20. Taking into consideration the short lifespan of O_2_^•−^ in an aqueous solution, we prepared a control sample that, according to the EPR study, did not form radicals, as follows: first, powdered KO_2_ was dissolved in a phosphate buffer adjusted with HCl to get a 20 mmol L^−1^ KO_2_ solution with a corresponding pH, then, 10 s later BMPO (final 10 mmol L^−1^) was added, and this mixture was applied at 1:1 volume ratio to pDNA solution. In accordance with the EPR data, the mixture prepared in this way had a negligible effect on the pDNA cleavage at all of the pH levels used (Figure 14).

## 4. Discussion

O_2_^•−^ and its derivatives are involved in radical signaling and, more significantly, in the development of diseases due to oxidative stress-mediated damage of the cellular structures [1,2]. To study the molecular mechanism of its biological actions, it is convenient to have a simple and reproducible source of O_2_^•−^. There are several methods to produce O_2_^•−^ [3]; however, some of them are not experimentally simple or are not suitable for biological experiments. Therefore, KO_2_ as a source of O_2_^•−^ was studied by the EPR spin trapping method without using non-biological components. As in our aqueous experimental system the O_2_^•−^ is highly reactive and spontaneously disproportionates within 10 s, this study was limited mostly to monitoring the secondary radicals (spin adducts) ^•^BMPO-OOH/^•^BMPO-OH, and did not study the production rates and fate of any primary radicals. Additionally, we are conscious of the pH-dependent reactivity of HO_2_^−^/H_2_O_2_ produced in the O_2_^•−^ disproportionation in the process of BMPO adduct generation.

The dissolution of KO_2_ by a phosphate buffer produces O_2_^•−^ and H_2_O_2_. O_2_^•−^ is known to form ^•^BMPO-OOH or ^•^BMPO-OH radicals, which are detectable by EPR and can be obtained by spectral simulation. From the good fit of the experimental spectra, it is obvious that the hfcc are suitable for spectral simulation. The hfcc values are in the range of data obtained under similar experimental conditions [4,6,8,9,11]. As our method distinguishes concentrations of the time-dependent changes of ^•^BMPO-OOH and ^•^BMPO-OH radicals in the same sample, it is appropriate for time-dependent studies of compound interactions with radicals. In ^•^BMPO-OOH and ^•^BMPO-OH nitroxide (aminoxyl) radicals, the unpaired electron is mostly localized on the N-O moiety and the hyperfine interactions with nitrogen and β-hydrogen nuclei dominate the EPR spectra (Table 1). Therefore, ^•^BMPO-OOH and ^•^BMPO-OH can be further studied as potential models of organic hydroperoxide and alcohol, respectively.

The dissolution of KO_2_ in a water solution (e.g., pH 7.4) produces O_2_^•−^ with a short lifetime (<ms) [3,7,23]. This was confirmed under our experimental conditions at pH 7.4, in which spin trap BMPO added 10–13 s after the preparation of the buffer/KO_2_ solution did not show any ^•^BMPO-adduct spectra. The same result was found when KO_2_ in DMSO was added to a pH 7.4 buffered water solution followed by BMPO [6].

Using the spin trap BMPO, we observed that the addition of a water solution to powder KO_2_ produced radicals, whose composition and intensity significantly depended on the pH level during the dissolution of KO_2_. As KO_2_ is a strong base, the addition of a buffered water solution (50 mmol L^−1^ phosphate buffer, pH 7.4) to powdered KO_2_ (final 40 mmol L^−1^) increased the pH to 10–11. There are two possible ways to decrease the pH of the mixture to the physiological value of 7.4. When pH was decreased to 6.5–8.5 through the addition of HCl to the prepared BMPO/KO_2_ mixture, ^•^BMPO-OH radicals, compared with ^•^BMPO-OOH, were mostly observed at pH ~7.3–10.3 (Figure 13). On the other hand, when the BMPO solution adjusted with HCl was added to the powdered KO_2_ to get a final physiological pH of ~6–8, ^•^BMPO-OOH prevailed over ^•^BMPO-OH (Figure 2b, Figure 4 and Figure 10). The ^•^BMPO-OOH/^•^BMPO-OH ratio calculated from the first recorded EPR spectrum was ≥20 (Figure 2b). As the first spectrum was recorded 100 ± 15 s after the BMPO–HCl solution was mixed with powdered KO_2_, it is very probable that the ^•^BMPO-OOH/OH ratio was even higher immediately after the sample preparation. Based on these data, it can be suggested that KO_2_ can be used as a source of O_2_^•−^ and ^•^BMPO-OOH, when it is ensured that during the dissolution of KO_2_, the pH is within the range of 6.5–8.0. This can be done by applying acid during the dissolution or by using a strong physiological buffer. However, as a significant amount of H_2_O_2_ is produced during the dissolution of KO_2_ by an aqueous buffer, its involvement in radical–compound interactions should be also taken into account.

It is supposed that the observed hydroxyl adduct, ^•^BMPO-OH, was mostly produced by pH- and time-dependent decomposition of ^•^BMPO-OOH, which was formed by trapping O_2_^•−^ during KO_2_ dissolution. This suggestion is supported by the time- and pH-dependent progressive increase in the relative concentration of ^•^BMPO-OH, which is more stable than ^•^BMPO-OOH, upon a progressive decrease in the ^•^BMPO-OOH relative concentration (Figure 2b and Figure 10). The significant proportion of the ^•^BMPO-OH component in the first recorded spectrum at pH ≥ 9.0 correlated well with published data [8]. However, the intensity of the ^•^BMPO-OH spectra transiently increased over time in the mixture of BMPO + H_2_O_2_ (pH 9.0 and 12.2; Figure S1), suggesting that the BMPO/H_2_O_2_ interaction contributed to the ^•^BMPO-OH component. Additionally, the decomposition of H_2_O_2_ into reactive oxygen species under alkaline media may promote the production of ^•^BMPO-OH [24,25,26,27]. As the present study was focused mainly on the practical preparation of ^•^BMPO-OOH, the mechanism of hydroxyl adduct formation was not studied in more detail.

Our previous study described the preparation of the ^•^BMPO-OOH spin adduct as a model for the study of organic hydroperoxide decomposition [6]. We used 10% DMSO (*v/v*) to prepare the ^•^BMPO adduct, which is useful for evaluating the antioxidant potency and effects of compounds insoluble in water on ^•^BMPO-OOH. In the present study, ^•^BMPO-OOH, as a potential model of hydroperoxide, was prepared without DMSO, and the antioxidant potency of compounds soluble in water, such as H_2_S, RSe-1, and RSe-2, was studied. As in our previous study with H_2_S in a 10% DMSO solvent [6,22], the water solution of H_2_S changed the ^•^BMPO-OOH/^•^BMPO-OH proportion and decreased the overall radical concentrations. RSe-1 and RSe-2 interacted with ^•^BMPO adducts and increased the radical proportion of ^•^BMPO-OH over ^•^BMPO-OOH in a time-dependent manner, even in the case when the total time-dependent ^•^BMPO adducts were similar to the controls. The detailed mechanism of the chemical interactions leading to changes in the concentration and/or ratio of ^•^BMPO-OOH/OH is not known at present and requires further examination.

The oxidation of DNA occurs through reactions with reactive oxygen species (ROS), e.g., HO^•^, O_2_^•−^, singlet oxygen (^1^O_2_), peroxynitrite (ONOO^−^), or H_2_O_2_, which are produced during endogenous biological processes [28,29,30]. Therefore, pDNA was used to test the biological effects of ^•^BMPO adducts. pDNA was significantly cleaved by low ^•^BMPO-OOH/^•^BMPO-OH concentrations, in which ^•^BMPO-OOH prevailed over ^•^BMPO-OH. BMPO, KO_2_, H_2_O_2_, and KO_2_ + H_2_O_2_ alone did not cleave pDNA, indicating that they were not involved in the pDNA cleavage effects of ^•^BMPO-OOH/^•^BMPO-OH. The mixture prepared for 10 s, which delayed the addition of BMPO to the buffer + HCl + KO_2_ solution, which did not form a ^•^BMPO adduct spectral signal, also did not cleave pDNA, confirming that neither the components of the mixture nor their nonradical products were able to cleave pDNA. Altogether, this implies that ^•^BMPO-OOH/OH radicals are responsible for pDNA cleavage. A detailed cascade of the chemical interactions leading to pDNA cleavage by ^•^BMPO-OOH/OH radicals is not known at the present and remains to be elucidated.

## 5. Conclusions

This study utilized KO_2_ as a source of O_2_^•−^ radical anions and the EPR spin trapping technique with BMPO to monitor the generation of the ^•^BMPO-OOH spin adduct and its conversion to ^•^BMPO-OH in a water solution without DMSO as a potential model for organic hydroperoxides and alcohols. The concentration and relative abundance of ^•^BMPO-OOH and ^•^BMPO-OH elucidated from the EPR spectra strongly depended on the procedure of the sample preparation and the pH value. In the pH range of 6.5–8.0, ^•^BMPO-OOH predominated over ^•^BMPO-OH. Moreover, the method also distinguished the time-dependent concentration changes of both ^•^BMPO adducts in the same sample, so it is appropriate for studies of time-dependent interactions of compounds with radicals. The ^•^BMPO-OOH/OH radicals cleaved the plasmid DNA. H_2_S and selenium-containing derivatives increased the proportion of ^•^BMPO-OH over ^•^BMPO-OOH radicals. This demonstrates that the presented approach can be used to study the biological effects of ^•^BMPO-OOH/OH radicals and the interactions of compounds with ^•^BMPO-OOH/OH.

## Figures and Tables

**Table 1:** The hfcc of BMPO spin adducts elucidated from simulations of experimental spectra measured in phosphate buffer solutions containing KO_2_. ^•^BMPO-OOH and ^•^BMPO-OH were simulated considering the presence of two conformers.

BMPO-Adduct	*a*_N_, mT	*a*_H_^β^, mT	*a*_H_^γ^, mT
^•^BMPO-OH(1)	1.433 ± 0.003	1.521 ± 0.005	0.074 ± 0.004
^•^BMPO-OH(2)	1.421 ± 0.004	1.264 ± 0.003	0.065 ± 0.002
^•^BMPO-OOH(1)	1.341 ± 0.003	1.198 ± 0.004	–
^•^BMPO-OOH(2)	1.340 ± 0.001	0.967 ± 0.006	–
^•^BMPO-CR	1.515 ± 0.008	2.077 ± 0.024	–
^•^BMPO-CO_2_^−^	1.490 ± 0.003	1.710 ± 0.012	–

## Data Availability

All findings and conclusions are based on the presented figures in the main text or in the Supplementary Materials. Original source files can be sent from the corresponding author, Dr. Karol Ondrias, upon request.

## Supplementary Materials

The following are available online at https://www.mdpi.com/article/10.3390/antiox10081286/s1, Procedures for preparation of EPR samples. Figure S1: Time- and pH-dependent EPR spectra of ^•^BMPO adducts of the 30 mmol L^−1^ BMPO in the presence of 1 and 10 mmol L^−1^ H_2_O_2_. Figure S2: Representative gels indicating the effects of BMPO−KO_2_ interaction on pDNA cleavage at various pH.
