# Supplementary material for: EPR Study of KO2 as a Source of Superoxide and •BMPO-OH/OOH Radical That Cleaves Plasmid DNA and Detects Radical Interaction with H2S and Se-Derivatives
Source: Antioxidants (Basel). 2021 Aug 13;10(8):1286. doi: 10.3390/antiox10081286 (PMC8389328; doi:10.3390/antiox10081286)
Supplement: Supplementary file 1 [file antioxidants-10-01286-s001.zip › antioxidants-1293304-supplementary.pdf]

antioxidants

MDPI

Supplementary Materials: EPR Study of KO_2 as a Source of Superoxide and •BMPO-OH/OOH Radical That Cleaves Plasmid DNA and Detects Radical Interaction with H_2S and Se-Derivatives

Anton Misak [1], Vlasta Brezova [2], Miroslav Chovanec [3], Karol Luspai [2], Muhammad Jawad Nasim [4], Marian Grman [1], Lenka Tomasova [1], Claus Jacob [4] and Karol Ondrias [1,*]

[1] Department of Molecular Physiology, Institute of Clinical and Translational Research, Biomedical Research Center, Slovak Academy of Sciences, Dúbravská cesta 9, 84505 Bratislava, Slovakia; anton.misak@savba.sk (A.M.); marian.grman@savba.sk (M.G.); lenka.tomasova@savba.sk (L.T.)

[2] Institute of Physical Chemistry and Chemical Physics, Faculty of Chemical and Food Technology, Slovak University of Technology in Bratislava, Radlinského 9, 81237 Bratislava, Slovakia; vlasta.brezova@stuba.sk (V.B.); karol.luspai@stuba.sk (K.L.)

[3] Department of Genetics, Cancer Research Institute, Biomedical Research Center, Slovak Academy of Sciences, Dúbravská cesta 9, 84505 Bratislava, Slovakia; miroslav.chovanec@savba.sk

[4] Division of Bioorganic Chemistry, School of Pharmacy, University of Saarland, D-66123 Saarbruecken, Germany; jawad.nasim@uni-saarland.de (M.J.N.); c.jacob@mx.uni-saarland.de (C.J.)

* Correspondence: karol.ondrias@savba.sk

Step-by-step information on sample preparation for EPR and pDNA cleavage measurements:

Procedures for preparation of EPR samples:

All EPR samples were prepared and measured at 37 ºC.

Procedure 1:

1.) Solution A.: (98 µl H_2O + 200 µl of 100 mmol L^{-1} sodium phosphate buffer; 200 µmol L^{-1} DTPA, pH 7.4 + 18 µl of 1 mol L^{-1} HCl) → pH 4.9 ± 0.3;

2.) Solution B.: Add 181 µl of Solution A into 0.5 mg of powdered KO_2. Mix it for 5 s;

3.) Incubate it for 10-13 s;

4.) Add 84 µl of (100 mmol L^{-1} BMPO in H_2O) into Solution B; → pH 7.4 ± 0.3.

Final concentrations in the sample: 40 mmol L^{-1} KO_2, 20 mmol L^{-1} BMPO, 50 mmol L^{-1} sodium phosphate buffer; 100 µmol L^{-1} DTPA, pH 7.4.

Procedure 2:

1.) Solution A.: 84 µl of 100 mmol L^{-1} BMPO solution in H_2O + (98 µl H_2O + 200 µl of 100 mmol L^{-1} sodium phosphate buffer; 200 µmol L^{-1} DTPA, pH 7.4 + 18 µl of 1 mol L^{-1} HCl) → pH 4.7 ± 0.3;

2.) Solution B.: Add 181 µl of Solution A into 0.5 mg of powdered KO_2. Mix it for 5 s; → pH 7.4 ± 0.3.

Final concentrations were the same as in Procedure 1.

Procedure 3:

1.) Prepare Solution B as in Procedure 2;

2.) Incubate 138.6 µl of the Solution B for 12 s;

3.) Add 1.4 µl of 10 mmol L^{-1} Na_2S in H_2O or 1.4 µl of 10 mmol L^{-1} RSe-1/ RSe-2 prepared in the phosphate buffer

Final concentrations were the same as in Procedure 1, besides 0.1 mmol L^{-1} Na_2S, RSe-1 or RSe-2.

Procedure 4:

1.) Solution A.: 84 µl of 100 mmol L^{-1} BMPO in H_2O + (98 µl H_2O + 200 µl of 100 mmol L^{-1} sodium phosphate buffer; 200 µmol L^{-1} DTPA, pH 7.4);

2.) Solution B.: Add 181 µl of Solution A into 0.5 mg of powdered KO_2. → pH 10-11;

3.) Incubate it for 30 s;

4.) Add 8 µl of 1 mol L^{-1} HCl → pH 7.4.

Final concentrations were the same as in Procedure 1.

Procedures for preparation of samples for pDNA cleavage studies:

The samples with pDNA were incubated 30 min at 37 ºC.

Procedure 5: Effect of KO_2 on pDNA alone or in the presence of H_2O_2:

1. Solution A.: 45 mmol L^{-1} sodium phosphate buffer, 90 µmol L^{-1} DTPA, pH adjusted with 1 mol L^{-1} HCl according to required final values (see next step);
2. Solution B.: 420 µl of solution A was added into 0.6 mg of powdered KO_2 → 20 mmol L^{-1} KO_2 in phosphate buffer pH 6.5, 7.4, 8.0 or 8.5 in final;
3. 10 s vortexing;
4. 10 µL of the mixture was added into 10 µL of pDNA (0.2 µg) in 5 mmol L^{-1} phosphate buffer, 10 µmol L^{-1} DTPA, corresponding pH → 10 mmol L^{-1} KO_2 in 25 mmol L^{-1} phosphate buffer, 50 µmol L^{-1} DTPA, pH 6.5, 7.4, 8.0 or 8.5;

   In the experiments involving H_2O_2, the solution of pDNA contained H_2O_2 (5 mmol L^{-1} in final).

Procedure 6: Effect of (BMPO+HCl) + KO_2 on pDNA assessed at different pH.

1. Solution A.: 45 mmol L^{-1} sodium phosphate buffer, 90 µmol L^{-1} DTPA, 10 mmol L^{-1} BMPO, pH adjusted with 1 mol L^{-1} HCl according to required final values (see next step);
2. Solution B.: 420 µl of solution A was added into 0.6 mg of powdered KO_2 → 20 mmol L^{-1} KO_2 and 10 mmol L^{-1} BMPO in phosphate buffer pH 6.5, 7.4, 8.0 or 8.5 in final;
3. 10 s vortexing;
4. 10 µL of the mixture was added into 10 µL of pDNA (0.2 µg) in 5 mmol L^{-1} phosphate buffer, 10 µmol L^{-1}, corresponding pH → 10 mmol L^{-1} KO_2 and 5 mmol L^{-1} BMPO in 25 mmol L^{-1} phosphate buffer, 50 µmol L^{-1} DTPA, pH 6.5, 7.4, 8.0 or 8.5.

Procedure 7: Effect of (BMPO+HCl) + KO_2 on pDNA assessed at pH 7.4.

1. Solution A.: 45 mmol L^{-1} sodium phosphate buffer, 90 µmol L^{-1} DTPA, 10 mmol L^{-1} BMPO, pH adjusted with 1 mol L^{-1} HCl according to required final values (see next step);
2. Solution B.: 420 µl of solution A was added into 0.6 mg of powdered KO_2 → 20 mmol L^{-1} KO_2 and 10 mmol L^{-1} BMPO in phosphate buffer pH 6.5, 7.4, 8.0 or 8.5 in final;
3. 10 s vortexing;
4. 5 mmol L^{-1} phosphate buffer (10 µmol L^{-1} DTPA) containing pDNA (0.2 µg) was adjusted with 0.5 mol L^{-1} NaOH (for pH 6.5) or 0.1/0.125 mol L^{-1} HCl (for pH 8.0/8.5) to standardize pH to final value of7.4

5. 10 μL of the mixture was added into 10 μL of pDNA (0.2 μg) in 5 mmol L^{-1} phosphate buffer, 10 μmol L^{-1}, pH adjusted according to the step 4→ 10 mmol L^{-1} KO_2 and 5 mmol L^{-1} BMPO in 25 mmol L^{-1} phosphate buffer, 50 μmol L^{-1} DTPA, pH 7.4.

Procedure 8: Effects of [(buffer+HCl) + KO_2] + BMPO on pDNA.

1. Solution A.: 50 mmol L^{-1} sodium phosphate buffer, 100 μmol L^{-1} DTPA, pH adjusted with 1 mol L^{-1} HCl according to required final values (see next step);
2. Solution B.: 378 μl of solution A was added into 0.6 mg of powdered KO_2 → 22 mmol L^{-1} KO_2 in 50 mmol L^{-1} phosphate buffer, 100 μmol L^{-1} DTPA, ~ pH 6.5, 7.4, 8.0 or 8.5;
3. 10 s vortexing;
4. 42 μL of 100 mmol L^{-1} BMPO was added into KO_2 solution (solution B) → 20 mmol L^{-1} KO_2 and 10 mmol L^{-1} BMPO in 45 mmol L^{-1} phosphate buffer, 90 μmol L^{-1} DTPA, pH 6.5, 7.4, 8.0 or 8.5;
5. 10 s vortexing;
6. 10 μL of the mixture was added into 10 μL of pDNA (0.2 μg) in 5 mmol L^{-1} phosphate buffer, 10 μmol L^{-1}, corresponding pH → 10 mmol L^{-1} KO_2 and 5 mmol L^{-1} BMPO in 25 mmol L^{-1} phosphate buffer, 50 μmol L^{-1} DTPA, pH 6.5, 7.4, 8.0 or 8.5;

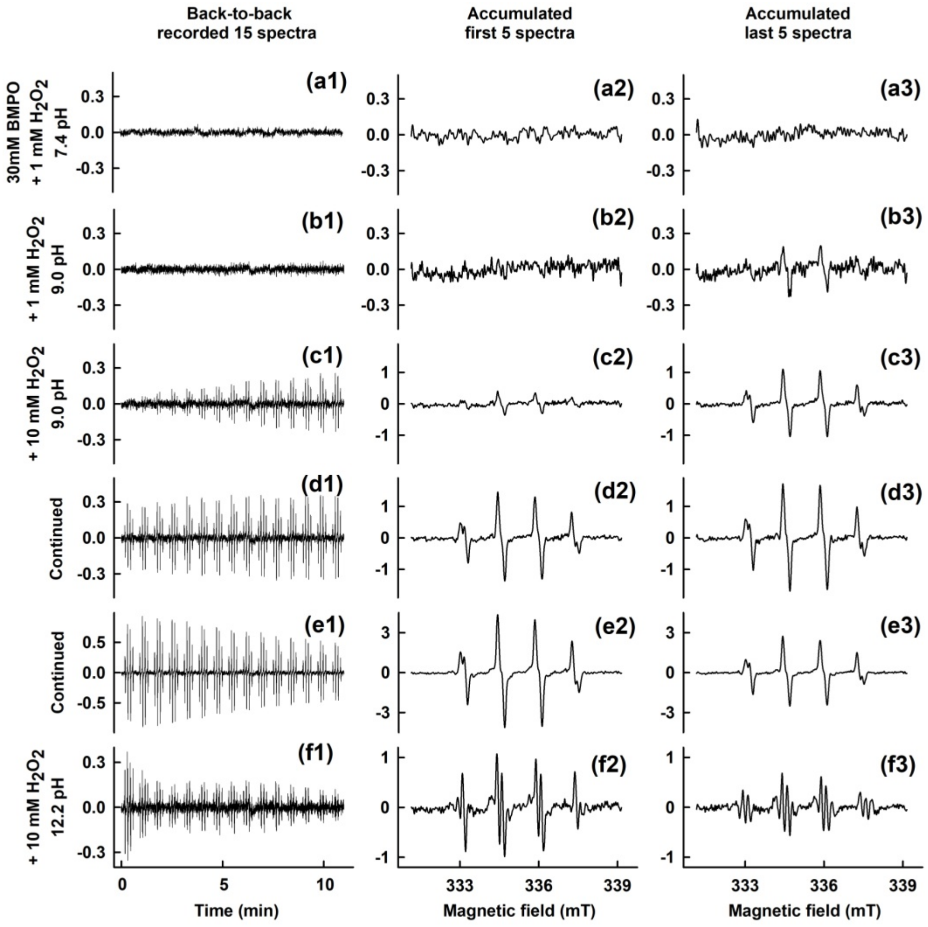

Figure S1. Time- and pH- dependent EPR spectra of $^{\bullet}$BMPO adducts of the BMPO in the presence of H_2O_2. (column **a1-f1**) Collection of 15 EPR spectra arranged back-to-back of the BMPO-adducts, each 42 s, with starting acquisition 100±15 s after sample preparation; (column **a2-i2**) first to fifth accumulated spectra and (column **a3-i3**) last five accumulated spectra. Buffer: 50 mmol L^{-1} sodium phosphate and 100 μmol L^{-1} diethylenetriaminepentaacetic acid (DTPA), pH 7.4 and adjusted by NaOH to 9.0 and 12.2. . EPR spectra of (**a1-a3)** 30 mmol L^{-1} BMPO in the presence of 1 mmol L^{-1} H_2O_2 at pH 7.4; (**b1-b3)** 30 mmol L^{-1} BMPO in the presence of 1 mmol L^{-1} H_2O_2 at pH 9.0; (**c1-c3)** 30 mmol L^{-1} BMPO in the presence of 10 mmol L^{-1} H_2O_2 at pH 9.0 and continued measurements (**d1-d3) and** (**e1-e3);** (**f1-f3)** 30 mmol L^{-1} BMPO in the presence of 10 mmol L^{-1} H_2O_2 at pH 12.2 EPR spectra were measured as described in (Misak et al. Antioxidants **2020**, 9, 918; doi:10.3390/antiox9100918).

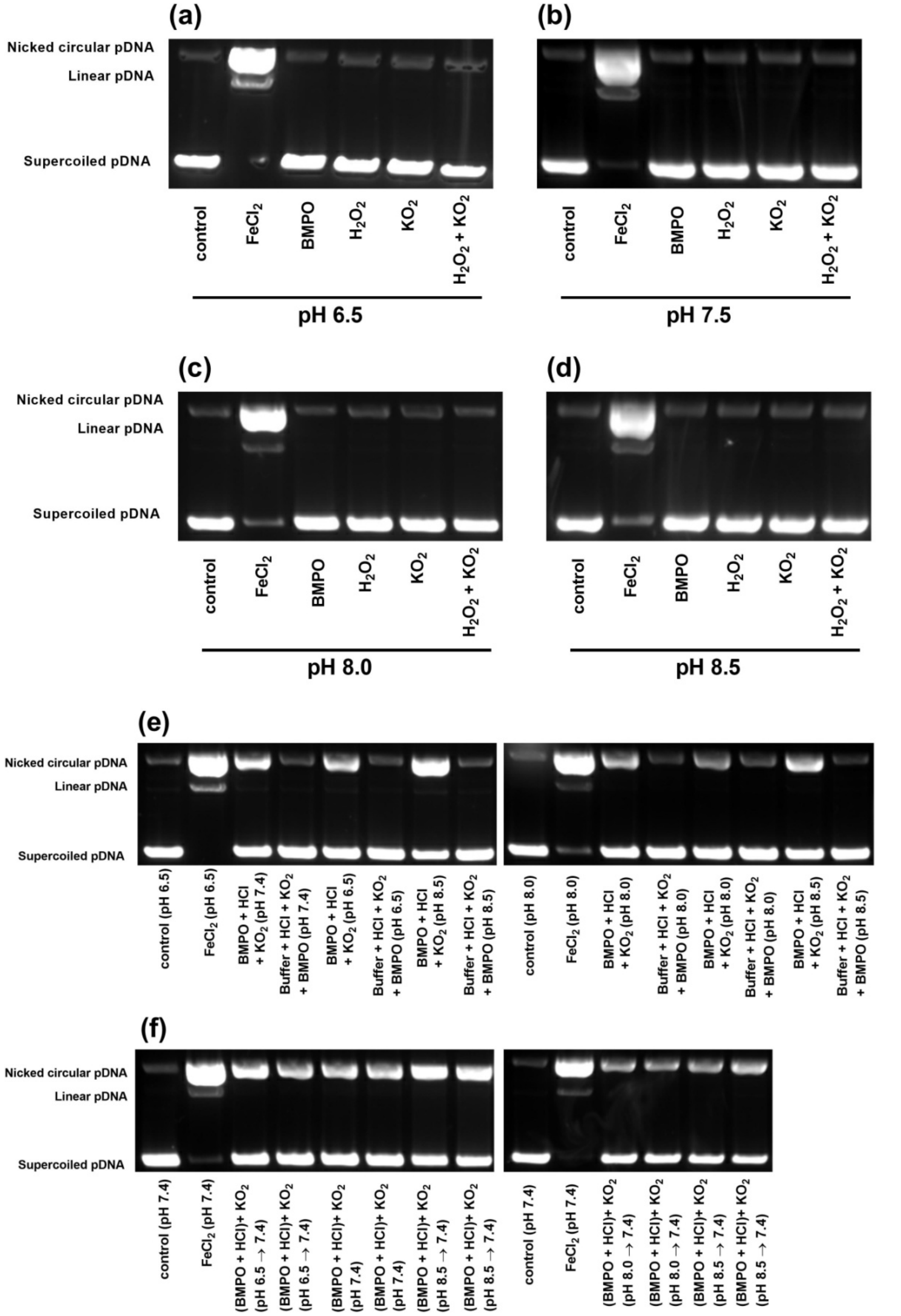

Figure S2. Representative gels indicating the effects of BMPO/KO_2 interaction on pDNA cleavage at various pH. Control samples containing: 5 mmol L^{-1} BMPO, 5 mmol L^{-1} H_2O_2, 10 mmol L^{-1} KO_2 or a mixture of 5 mmol L^{-1} H_2O_2 with 10 mmol L^{-1} KO_2 in 25 mmol L^{-1} sodium phosphate (100 µmol L^{-1}) at pH 6.5 **(a)**, pH 7.4 **(b)**, pH 8.0 **(c)** and pH 8.5 **(d)** are shown. The effect of 5/10 mmol L^{-1} BMPO/KO_2 mixture at various pH **(e)** was measured in two modes: in the first one KO_2 was dissolved in pre-prepared (BMPO + HCl) solution or in the second one KO_2 was dissolved in (Buffer+HCl) and

10 seconds later BMPO was added. To compare the pDNA cleavage induced by 5/10 mmol L^{-1} BMPO/KO_2 mixture at similar pH conditions **(f),** the sodium phosphate buffer pH 6.5, 8.0 or 8.5 was adjusted to pH 7.4. The strong cleavage induced by 150 μmol L^{-1} $FeCl_2$ is shown for comparison. Final concentration of pDNA was 0.2 μg in 20 μL. The top bands on the gel correspond to the nicked circular form of pDNA. Besides single-stranded cleavage of the supercoiled pDNA generating the nicked circular form, double-stranded cleavage events creating linear form of pDNA were also observed even though only rarely. Principally, the linear form of pDNA can be generated from supercoiled circular form in two ways. As a consequence of random distribution of single-stranded nicks along the pDNA giving rise also to a situation when two nicks on the opposite DNA strands are localized in close vicinity, usually being ~ 10 to 20 bp apart. Such structure is thermodynamically unstable and causes local dissociation of pDNA strands and linearization of pDNA. In addition, for example as a result of action of Fe(II), binding of Fe(II) ion to pDNA backbone mediates multiple pDNA strand nicking events and this action linearizes pDNA. The bottom bands correspond to the (circular) supercoiled form of pDNA, and the less intense bands appearing above, in the case of $FeCl_2$, represent the linear form of pDNA.
